# Genetic cargo and bacterial species set the rate of vesicle-mediated horizontal gene transfer

**DOI:** 10.1038/s41598-017-07447-7

**Published:** 2017-08-18

**Authors:** Frances Tran, James Q. Boedicker

**Affiliations:** 1 University of Southern California, Department of Biological Sciences, Seaver Science Center (SSC) 212, 920 Bloom Walk, Los Angeles, CA 90089 USA; 2 University of Southern California, Department of Physics and Astronomy, Seaver Science Center (SSC) 212, 920 Bloom Walk, Los Angeles, CA 90089 USA

## Abstract

Most bacteria release extracellular vesicles (EVs). Recent studies have found these vesicles are capable of gene delivery, however the consequences of vesicle-mediated transfer on the patterns and rates of gene flow within microbial communities remains unclear. Previous studies have not determined the impact of both the genetic cargo and the donor and recipient species on the rate of vesicle-mediated gene exchange. This report examines the potential for EVs as a mechanism of gene transfer within heterogeneous microbial populations. EVs were harvested from three species of Gram-negative microbes carrying different plasmids. The dynamics of gene transfer into recipient species was measured. This study demonstrates that vesicles enable gene exchange between five species of Gram-negative bacteria, and that the identity of the genetic cargo, donor strain, and recipient strain all influence gene transfer rates. Each species released and acquired vesicles containing genetic material to a variable degree, and the transfer rate did not correlate with the relatedness of the donor and recipient species. The results suggest that EVs may be a general mechanism to exchange non-specialized genetic cargo between bacterial species.

## Introduction

Microorganisms possess complex abilities to transfer genetic material through horizontal gene transfer (HGT), fundamentally shaping genetic landscapes and affecting biological functions^1–4^. The capacity for DNA exchange in bacteria and the plasticity of their genetic material has amplified the rate of adaptation and evolution across species and has allowed for stability and growth of complex microbial ecosystems in a multitude of environments^5–8^. The ability of diverse bacterial populations to share genes supports cooperation and survival^9, 10^, and, in specific cases, contributes to the emergence and spread of antibiotic resistance and pathogenicity^11, 12^. Within heterogeneous populations, DNA transfer occurs within and between different species^13–18^. Polz *et al*. describe continuous and widespread horizontal gene transfers established through local gene networks^19^. Known limitations of the standard gene transfer pathways of transduction, transformation, and conjugation, and the extent of interspecies gene exchange within wild populations suggests there maybe alternative mechanisms of gene transfer^20, 21^.

Our current understanding of HGT consists of three well-described mechanisms to exchange genetic material between bacteria: transformation, conjugation, and transduction^2–4, 6, 22^. Transformation is the uptake of extracellular DNA^6, 23–27^. Free DNA is common in nearly all environments as the result of active excretion by living cells and cell death and lysis, therefore, recipient uptake is the main barrier to gene transfer by transformation. To acquire free DNA, cells must be in a state of competence, which is both transient and affected by many factors. Natural competence is often tightly regulated, involving 20 or more proteins^23, 28–31^. The number of species capable of natural transformation appears to be small, with rough estimates of 1% of bacterial species. Conjugation is mediated by cell-to-cell contact during which a pore directs transfer of DNA. Conjugative transfer systems are associated with and depend on plasmids that code for the necessary proteins to facilitate DNA exchange^14, 32–38^. The conjugative apparatus also allows for the transfer of other genetic material, known as mobile genetic elements. In general, horizontal gene transfer through conjugation is specific to a small set of specialized gene products, although some broad range conjugative systems have been identified^39–41^. The third form of HGT relies on bacteriophages that transfer DNA through infection. Bacteriophages package their own and non-phage DNA, in some cases taking up to 100 kilobases of “extra” genes with them to infect new cells^42^. As described by Marks and Sharp, cell surface interactions with bacteriophages limit host specificity^7, 42–45^. Although these known mechanisms enable gene flow within microbial ecosystems, these three mechanisms of horizontal gene transfer present barriers to gene exchange, e.g. limited genetic cargo in the case of conjugation, limited recipients in the case of transformation, and limited donor-recipient pairs in the case of transduction^20, 46^. Here we explore the properties of an emerging gene transfer mechanism, vesicle-mediated gene exchange.

Recent studies have uncovered a newly identified mechanism for DNA transfer utilizing ubiquitously produced extracellular vesicles^47^. Nearly all observed bacteria, including both Gram-negative and Gram-positive bacteria, secrete spherical structures known as EVs^48–55^. These nanovesicles, 20–300 nm in diameter, are produced and released during growth. Bacterial extracellular vesicles contain both periplasmic and cytoplasmic components, however, many aspects of vesicle biogenesis and the regulation of vesicle composition remain unclear^51, 56, 57^. Bacterial extracellular vesicles have been shown to serve a variety of functions in intra- and interspecies microbial communities^58–62^, and participate in protein and signal exchange between microbes and hosts^62–66^. These vesicles coordinate many forms of intercellular communication and facilitate the exchange of small molecules, proteins, and nucleic acids, including RNA and DNA^65–68^.

Vesicle-mediated transfer has been identified as an additional form of gene exchange. Intraspecies vesicle-mediated gene transfer has been reported in *Escherichia coli*, *Acinetobacter baumannii*, *Acinetobacter baylyi* and *Pseudomonas aeruginosa* ^69–72^. Recent work by Fulsundar *et al*. demonstrated interspecies gene transfer from *A*. *baylyi* EVs to *E*. *coli* cells. The unknown specificity and limitations of vesicle-mediated gene transfer make it difficult to gauge its importance to gene exchange in the wild. Extracellular vesicles are found in all studied species and the loading of DNA has been shown to be wide-spread^50, 57, 73^. We currently lack an understanding of DNA loading specificity, whether or not it is regulated, and what parameters determine the rate of vesicle uptake. Because vesicle exchange has been observed between distantly related cells, this suggests it may enable pervasive, low-barrier gene exchange. Here we explore the hypothesis that vesicle-mediated gene transfer is able to package multiple types of genetic material and that the species involved determine the rate of interspecies gene exchange.

## Results

### Bacteria package multiple types of plasmids into extracellular vesicles

**Figure 1:**
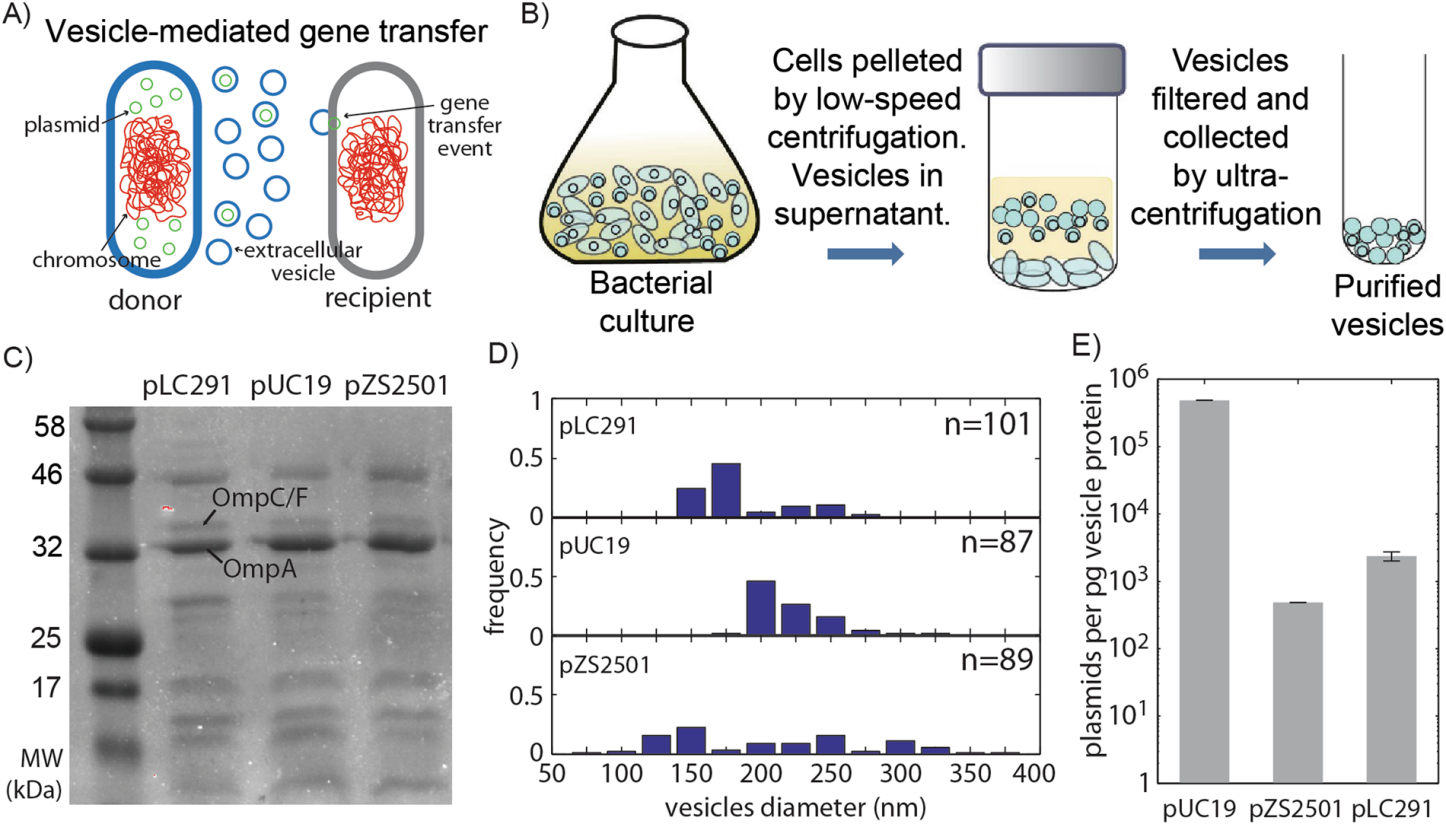
The impact of plasmid identity on the production and packaging of plasmid-containing vesicle. (**A**) Vesicle-mediated gene transfer. Donor cells load plasmid DNA into EV vesicles that can be acquired by a recipient cell. (**B**) Purification of EVs from liquid culture of bacterial cells through ultracentrifugation of cell-free supernatant. (**C**) 10% SDS-PAGE gel stained with Coomassie Blue showing concentration of outer membrane proteins, OmpA and OmpC/F, from EVs. (**D**) Distribution of EV diameters measured by dynamic light scattering. (**E**) Vesicle DNA content per pg of vesicle protein quantified by qPCR. P-value for all paired plasmid comparisons <0.001. Error bars signify standard deviation.

Incorporation of bacterial plasmids into EVs has been previously observed^70–73^, but it is unclear if plasmid characteristics influence packaging. To examine the effects of plasmid identity on vesicle loading, we harvested vesicles using ultracentrifugation of the cell-free supernatant from stationary phase liquid cultures of *Escherichia coli* (Fig. 1B) carrying plasmids with different replication origins: pLC291, pUC19 and pZS2501 (Table S1). Purified EVs were filter sterilized to remove all bacterial cells and subsequently treated with DNase I to remove free DNA. Both pLC291 and pUC19 are high-copy number plasmids, with origins RK2 and pMB1 respectively^74, 75^. The RK2 origin was originally isolated from *Klebsiella aerogenes* ^76^, and pMB1 was originally isolated form *E*. *coli* ^77^. pZS2501 is a low-copy plasmid with the pSC101 origin^78^, originally isolated from *E*. *coli* ^79^. All three plasmids were demonstrated to be loaded into EVs from *E*. *coli*, see Fig. S1.

We characterized EVs isolated from cells carrying each of the three plasmids. The production of EVs was quantified, similar to previously used methods^80, 81^, by SDS-PAGE stained with Coomassie blue to measure outer membrane protein concentration (Figs 1C and S2). The total amount of EVs produced was similar for hosts containing each of the plasmids, vesicles contained roughly 0.05 mg of membrane protein. See Supplementary Materials for calculations to relate membrane protein content to vesicle number. Vesicle yield measured by protein concentration was confirmed using the BioRad Bradford assay (Fig. S3). Total protein was slightly higher when measured by Bradford which is likely caused by cellular debris proteins. We also compared vesicle production between plasmids using fluorescent lipophilic dye FM4–64 to measure relative EV production confirming nearly identical vesicle production by all three plasmids (Fig. S4). The size distribution of the vesicles was measured using dynamic light scattering^23, 30, 81^. Vesicle diameters were between ~100–300 nm for all three plasmids. Although vesicles harvested from hosts containing pZS2501 having a wider size distribution, each have average diameters of roughly 0.2 µm (Fig. 1D). DNA loading into vesicles was measured using quantitative PCR (Fig. 1E). Vesicles were lysed by boiling, and the amount of DNA in 0.001 μg of vesicles was quantified using primers designed around a ~200 bp region of each plasmid origin, see Table S3. Standard curves made using mini-prepped purified plasmids were used to calculate plasmid copy numbers (Fig. S5). The low copy number plasmid pZS2501 had the lowest loading density, with only 0.49 × 10^3^ copies per pg of vesicle protein. pLC291 and pUC19 had 2.58 × 10^3^ and 482.68 × 10^3^ copies of plasmid per pg of vesicle protein, respectively. Taken together, our findings demonstrate that *E*. *coli* is able to load multiple types of plasmids into EVs and suggest that plasmid identity has an effect on vesicle size and gene loading.

### Vesicle-mediated gene transfer is affected by plasmid identity

**Figure 2:**
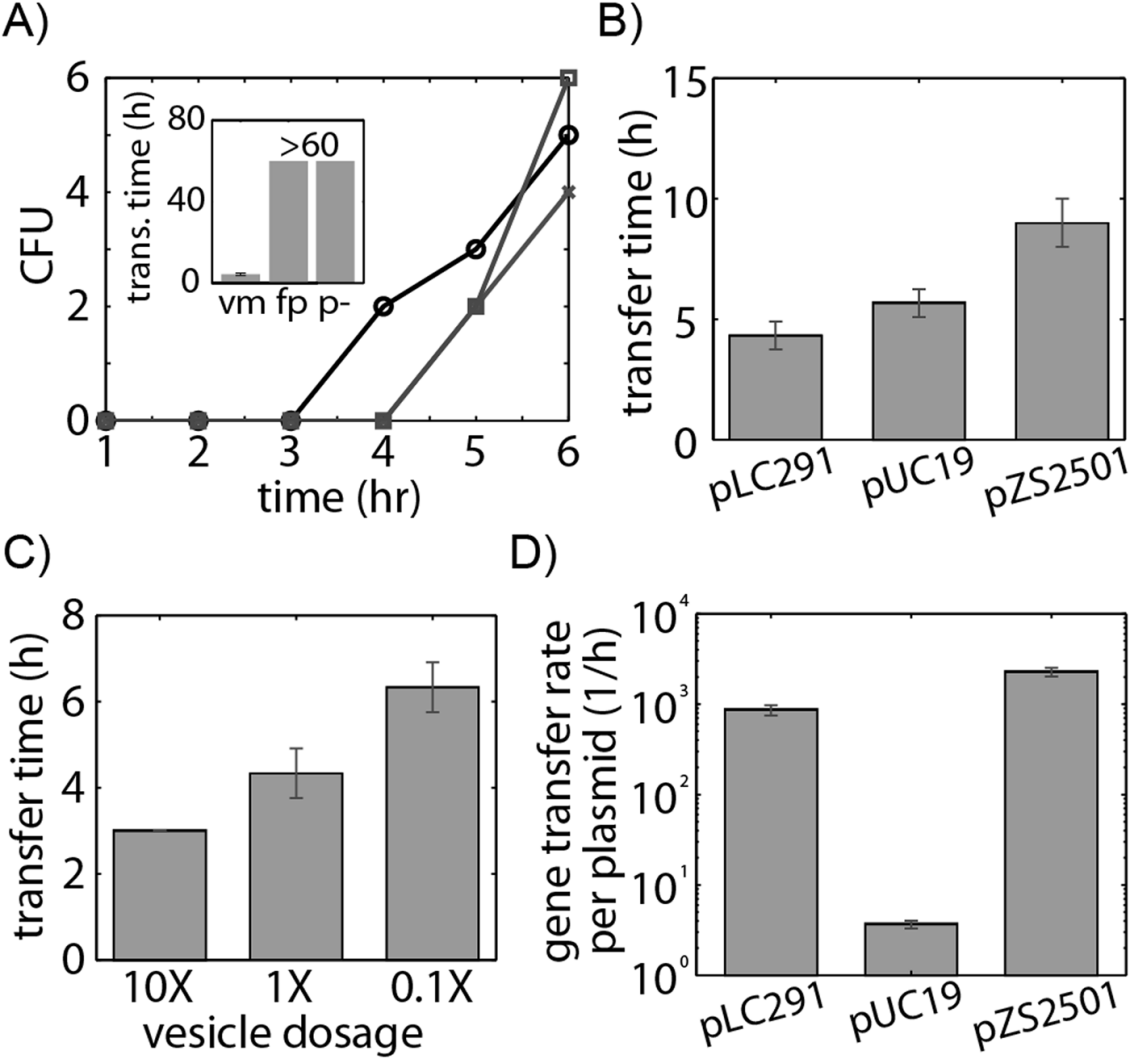
EVs facilitate HGT of multiple plasmids at a rate dependent on plasmid origin. (**A**) Purified EVs loaded with pLC291 were added to a recipient strain culture. Over time, the gain of antibiotic resistance was monitored by selective plating. The three curves show replicate experiments. The transfer time is the average time point at which resistant colonies were first observed. Inset shows recipient cells receiving either free plasmids (fp) or vesicles harvested from cells not containing the plasmid (p-) did not gain resistance by t = 60 h, whereas vesicle-mediated transfer (vm) occurred at 3.7 h. (**B**) Transfer time for EVs containing the plasmids pLC291, pUC19, and pZS2501. All pairs of transfer times have p-values < 0.05. (**C**) Gene transfer assay for three doses of vesicles containing pLC291. 1X corresponds to EV solution with 0.01 mg of characteristic outer-membrane protein (20% of total harvested). (**D**) Transfer rates for each plasmid were normalized by the number of plasmids per vesicle. Transfer rates have p-values < 0.001. Error bars signify standard deviation.

We next investigate the role of plasmid type on gene transfer rates. We isolated EVs from *E*. *coli* containing one of three plasmids, pLC291, pUC19 or pZS2501, as described above. Based on established protocols to measure gene transfer rate by transformation and transduction^7, 25^, we grew *E*. *coli* recipient cells to early log phase, OD_600_ 0.2, and added 0.01 mg purified vesicles containing plasmid. Over time, 200 µL aliquots of the culture were transferred to LB agar with antibiotic to detect the transfer of the plasmid resistance marker to the recipient strain, enabling detection of resistant cells at densities above ~5 CFU/mL. As shown in Fig. 2A, after several hours antibiotic resistance was detected in the recipient strain. The inset to Fig. 2A shows that neither the addition of free DNA to the recipient culture nor purified vesicles from *E*. *coli* cultures without plasmids resulted in gain of antibiotic resistance (measurements taken for 60 h), demonstrating the essential role of EVs in gene transfer. Vesicle solutions were also treated with DNase I to remove genetic material outside of EVs. Resistant colonies were verified to have acquired plasmid DNA by re-streaking and running diagnostic colony PCR. We identify time to transfer based on the first appearance of antibiotic resistant colonies (Fig. 2B). Plasmid pLC291 transfers the fastest, after 4.5 hours, followed by pUC19 after 5.7 hours and pZS2501 after 8 hours. Transfer rates were not significantly (p = 0.62) influenced by choice of resistance marker, see Fig. S6. To address possible effects of plasmid size to transfer rates, we constructed plasmids with the same 3500 bp size varying only the origin of replication (Fig. S7). This data confirms the effects on transfer rates are dominated by plasmid identity regardless of plasmid size.

We next show, using EVs packed with pLC291, that the transfer time is proportional to the number of vesicles added at the beginning of the gene transfer measurement. We use *E*. *coli* EVs packed with pLC291 as our donor at 0.1 mg, 0.01 mg, and 0.001 mg concentrations into *E*. *coli* recipient cells and demonstrate that the transfer of pLC291 via EVs is dose dependent (Fig. 2C).

Even vesicle dosages of 0.001 mg generated measureable gene transfer within 12 h. As detailed in the Supplementary Materials, we estimate that vesicles containing 0.01 mg of Omp proteins contain 1.3 × 10^10^ individual vesicles. These calculations take into account the typical density of Omp proteins in the outer membrane, 6 × 10^−9^ µg OmpA/µm^2^ of membrane^82^, and the size of the vesicle. Stationary phase cultures of the donor contained 0.4 vesicles/cell. Vesicle uptake experiments were conducted at 0.8 vesicles/cell, similar to vesicle dosages found in stationary phase cultures. Figure 2C shows that transfer time for vesicles containing pLC291 were about 1.5 hours slower when vesicle dosage was reduced by a factor of 10. If this scaling holds for both reduction in vesicle number and recipient cell number, we estimate that a single vesicle transfer event would occur every 7.3 hours for 1 mL of stationary phase culture.

In Fig. 2D, we separate the effects of plasmid identity from plasmid copy number by normalizing rate of vesicle-mediated transfer by plasmid copy numbers reported in Fig. 1E. The transfer rate is defined as the inverse of the transfer time. Accounting for differences in plasmid loading, pZS2501 transfers the fastest followed by pLC291 and then pUC19, despite pUC19 having the highest plasmid copy number and plasmids per vesicle. Vesicle-mediated gene transfer rates are influenced by both the EV concentration and characteristics of the DNA cargo, including the plasmid origin as shown here.

### Plasmid packaging did not depend on the donor strain

**Figure 3:**
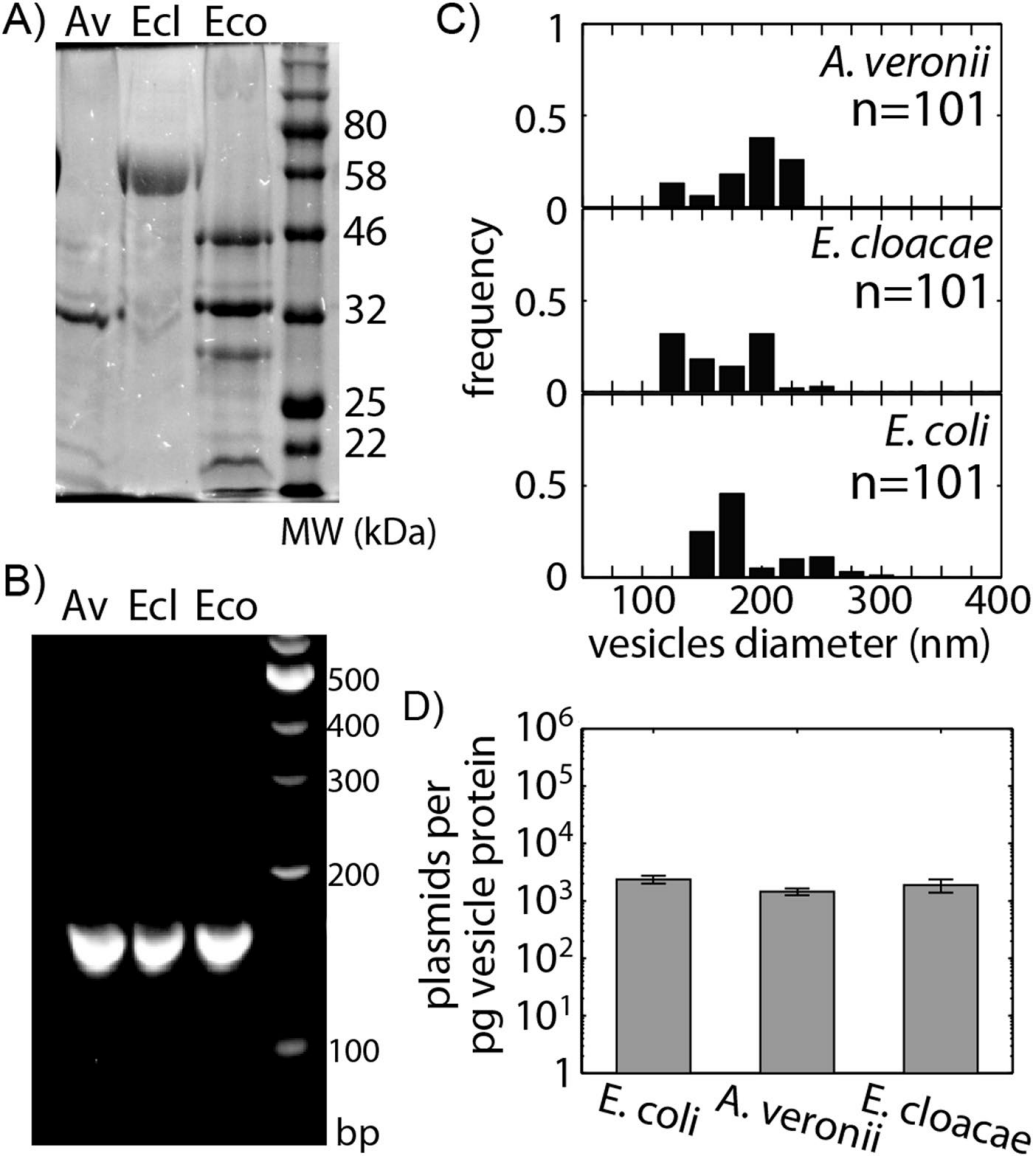
Variation of EVs and plasmid packaging is less dependent on donor species. (**A**) The production of EVs from *A*. *veronii* (Av), *E*. *cloacae* (Ecl), and *E*. *coli* (Eco), are measured using SDS-PAGE to quantify protein concentration. EV production is similar across species. (**B**) Packaging of pLC291 is confirmed using PCR amplification from purified vesicles. (**C**) Size distributions of EVs measured by dynamic light scattering shows similar diameters of vesicles harvested from all three species. (**D**) Vesicle DNA content quantified by qPCR was used to calculate plasmid copy number per pg of vesicle protein. P-values ≤ 0.0002. Error bars signify standard deviation.

We next investigate the potential for EVs being released from different bacterial species to load plasmid DNA. Three bacterial species, *Aeromonas veronii*, *Enterobacter cloacae* and *Escherichia coli* are used as EV donors. The first two species, *A*. *veronii* and *E*. *cloacae*, are wild isolates while *E*. *coli* is the laboratory strain used in Figs 1 and 2. To look at EVs production and plasmid loading, broad-host range plasmid pLC291 with a kanamycin resistance selection marker was transformed into donor strains by electroporation. Extracellular vesicles from all three species carrying pLC291 were isolated from late-stationary phase liquid cultures as described above. The amount of outer membrane proteins in the harvested vesicles were quantified using SDS-PAGE (Fig. 3A). EVs are produced at relatively similar concentrations as conferred by membrane protein concentration. Each species produced about 0.05 mg of vesicle protein. The most abundant membrane proteins are seen at about 30, 60, and 45 kDa for *A*. *veronii*, *E*. *cloacae* and *E*. *coli* respectively^83, 84^. PCR targeting a 300 bp region of the plasmid was used to verify pLC291 loading into vesicles purified from all three species, (Fig. 3B). All three species showed a similar distribution of EVs diameters, (Fig. 3C). As in Fig. 1D, qPCR was used to quantify plasmid copy number per 1 pg of outer-membrane protein in purified donor vesicles. All three species package pLC291 into their EVs at similar concentrations ranging from 1350–2580 plasmids per pg of outer membrane protein (Fig. 3D).

### Rates of vesicle-mediated gene transfer are dependent on species but not relatedness

We next examine the potential for EVs to facilitate interspecies gene transfer. Interspecies gene transfer relies on the capacity of recipient species to take-up and maintain horizontally acquired DNA. Recipient cells actively try to degrade foreign DNA using restriction enzymes, and plasmid expression and replication are additional barriers of gene transfer^22^. These barriers of plasmid maintenance upon introduction to the recipient species contribute to the dependence of HGT on the relatedness of the donor and recipient species^17, 85^. To focus on how relatedness influences the uptake of EVs containing plasmids, for interspecies transfer experiments we use plasmid pLC291 with broad-host range origin RK2 which is part of the IncP-1 plasmid group from *E*. *coli*. The original RK2 plasmid was isolated from *Klebsiella aerogenes* infections of burn patients^76^, and this origin was later developed for use in *Methylobacterium extorquens* ^75^. In control experiments all recipient strains were capable of maintaining the plasmid. All recipient strains gained resistance within 20 min of electroporation (Fig. S8), demonstrating the time needed to express resistance genes did not strongly influence the measured differences in vesicle-mediated gene transfer times.

**Figure 4:**
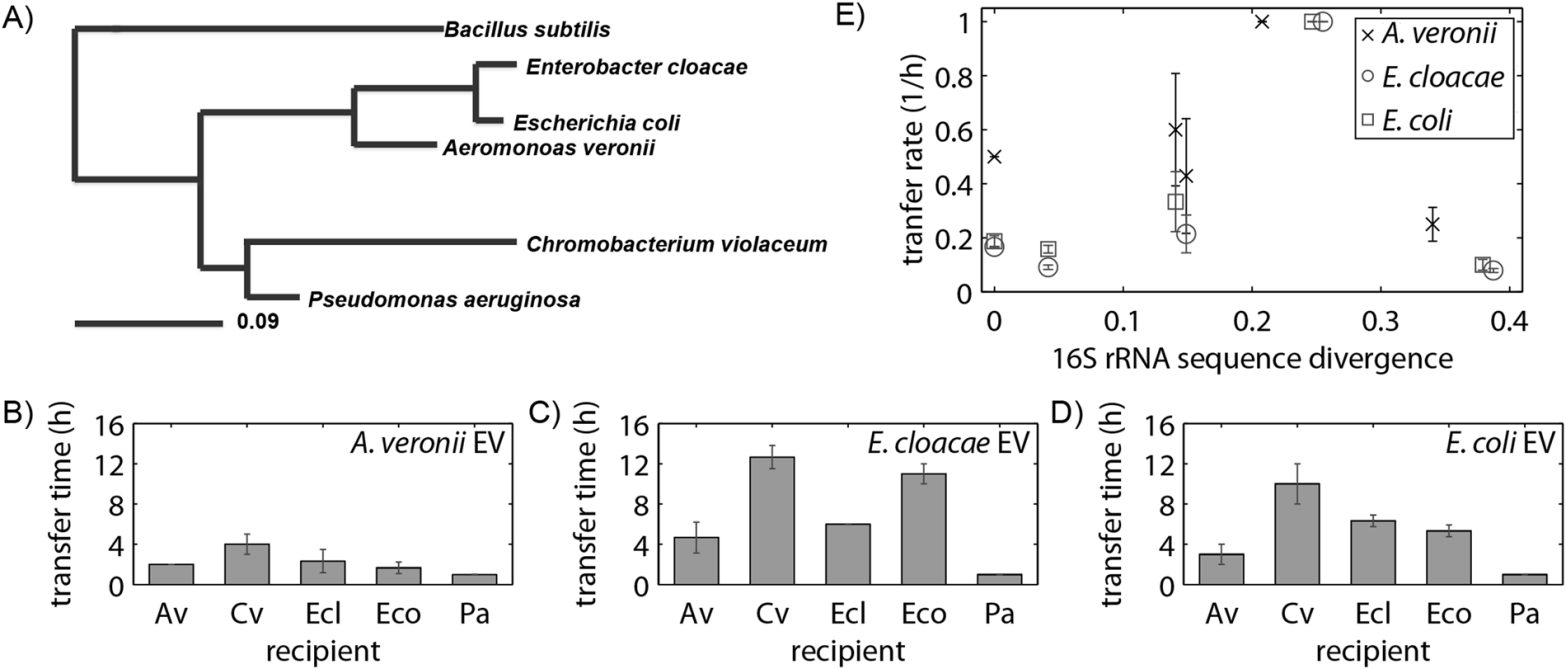
Donor and recipient species influence rates of vesicle mediated gene transfer. (**A**) Relatedness of recipient species based on 16S rRNA sequence. Donor EVs packed with pLC291 from donor strains *A*. *veronii* (**B**), *E*. *cloacae* (**C**) and *E*. *coli* (**D**) were added to recipients strains *A*. *veronii*, *C*. *violaceum*, *E*. *cloacae*, *E*. *coli*, and *P*. *aeruginosa*. The time to plasmid transfer was measured by selective plating as in Fig. 2. (**E**) The transfer rate was not correlated with the relatedness of the donor and recipient strains, as measured by divergence of the 16S rRNA sequence. Error bars signify standard deviation from 4 replicates.

Vesicles were isolated from *A*. *veronii*, *E*. *cloacae* and *E*. *coli* containing the plasmid pLC291. We selected 5 recipient species that range in their relatedness based on 16S rRNA sequencing (Fig. 4A). Other studies of HGT mechanisms have shown transfer rates to by highly affected by relatedness of bacterial species^25, 85^. Therefore, to investigate whether EV-mediated gene transfer is influenced by relatedness, donor vesicles from three strains were added to cultures of the 5 recipient species, *A*. *veronii*, *E*. *cloacae*, *E*. *coli*, *Chromobacterium violaceum* and *Pseudomonas aeruginosa*. The time to plasmid transfer was measured for each donor/recipient pair, revealing strong species dependence on gene transfer (Fig. 4B–D). *A*. *veronii* transfers pLC291 via EVs in less time than the other donor strains. There is also significant variability in recipient acquisition of EV carrying plasmids. *P*. *aeruginosa* gains antibiotic resistance via plasmid uptake in the shortest time regardless of the donor species. The data also suggests that *A*. *veronii* acquires pLC291 from EVs on a slightly faster time scale than the other three recipients, *C*. *violaceum*, *E*. *cloacae* and *E*. *coli*.

To quantify if gene transfer times correlate with the relatedness of the donor and recipient species, the time to transfer was plotted against the 16S rRNA divergence, as shown in Fig. 4E, revealing no clear trend in relatedness and transfer time. Our findings indicate that certain species of bacteria produce DNA packed vesicles that are more readily transferred than others and that specific species have a greater capacity to uptake DNA in vesicles.

## Discussion

Horizontal gene transfer enables microbes to rapidly evolve and adapt to complex and constantly changing environments. Large genetic modifications attributed to ubiquitous gene transfer have been found to be widespread across populations of bacteria demonstrating its importance and pervasive nature. Three well-studied, canonical mechanisms, transformation, transduction, and conjugation, contribute significantly to the genetic diversity that is shared between species. Yet, the level and extent to which different HGT events shape microbial genomes is still being studied^12, 22^. It is probable that other mechanisms contribute to the general abundance of gene exchange, given known barriers to gene exchange in these textbook exchange pathways^20^. In this study, we look at the role of a recently discovered mechanism for HGT, vesicle-mediated transfer, and its capacity to facilitate intra- and interspecies gene exchange^48–50, 62^. Membrane vesicles have been previously reported to carry DNA and have been demonstrated to mediate HGT within species and between one pair of species^57, 70, 73^. Here we examine the transfer of multiple types of plasmids between several species of Gram-negative bacteria, finding that plasmid type influences both DNA packaging and transfer rates, and that gene transfer rate depends on the identity of the donor and recipient species.

EVs from *E*. *coli* are capable of packaging several non-specialized plasmids. Unlike conjugation, which requires specific origins of transfer and the conjugation machinery conferred on the plasmid^33^, we demonstrate plasmids with three different origins encoding no genes or sequences specialized for gene transfer were successfully loaded into harvested EVs. The ability to package a range of plasmids suggests the capacity for vesicle-mediated gene transfer to widely contribute to gene transfer within populations, although not all plasmids transferred at the same rate.

The efficiency of loading of plasmids into vesicles was strongly dependent on plasmid identity. pUC19 had 2–3 orders of magnitude more plasmid per vesicle than both pZS2501 and pLC291. We use Fig. S5 to we estimate the loading percentages of each plasmid type into vesicles, and find an average of 3.62, 0.18, and 0.04 plasmids loaded per vesicle for pUC19, pLC291, and pZS2501 respectively. Estimated loading percentages follow the ranking of plasmid copy number, the copy number of pUC19 is twice that of pLC291 and almost 10 times that of pZS2501, see Table S1, however plasmid loading was not directly proportional to copy number. Characteristics such as plasmid size, protein binding, or the location of each plasmid type within the cell may influence vesicle loading. Recent work has demonstrated complex systems for plasmid organization within cells that are influenced by cell cycle and growth rate^85, 86^. Studies in extracellular vesicle production in a range of bacterial species have also demonstrated the influence of cell cycle on vesicle production^87^. More work is needed to understand the rules for plasmid packaging into vesicles. It remains an open question whether or not plasmid packaging is a random process, or conversely if plasmids or bacteria evolved mechanisms for increased packaging efficiencies.

Plasmid features also play a role in the rate of gene transfer (Fig. 2B and C). Surprisingly when adjusted for plasmid loading, the transfer rate of plasmids containing pUC19 was more than 100 times slower than the other plasmids. It is unclear how genetic cargo would impact transfer rates. Potentially, genetic cargo modulates vesicle properties related to interactions within donor strains and can also affect recipient uptake. One complication in interpreting transfer rates is that the distribution of plasmid copy numbers within the vesicles is unknown. Although we estimate an average loading of 3.6 plasmids per vesicle in the case of pUC19 compared to 0.04 plasmids per vesicle with pZS2501, some vesicles might be empty and others might contain many plasmids. It is possible the plasmid origin plays a role in transfer times due to the dependence of transfer on both plasmid acquisition and maintenance, however, all three plasmids were stably maintained with the *E*. *coli* host. Also the selection marker did not significantly influence transfer times, see Fig. S6. Although the biological significance is yet to be defined, our results point to vesicle mediated horizontal gene transfer to be dependent on genetic content.

To what extent vesicle-mediated gene transfer contributes to gene exchange in wild populations is still unresolved, although these results suggest that the rate of gene transfer via vesicles is not prohibitively slow. Stationary phase cultures reached ratios of nearly one vesicle for every cell, or 0.25 μg vesicle protein per mL of culture. At these ratios the time to a gene transfer event should be on the order of hours for 1 mL of culture (see Supplementary information for details). It should be noted that vesicles harvested from cells in other growth phases may have different properties and dynamics^88, 89^. The details of vesicle biogenesis are still an active area of research^61, 90, 91^. The time needed for gene transfer determines the number of recipient cells within a population, predicted to be a critical factor in the fixation of horizontally transfer genes^92^. The selection coefficient of transferred genes also plays a critical role, especially when gene transfer events are rare^92^. Understanding the cellular characteristics that set both selection criteria and transfer rates of a given gene will help predict the impact of vesicle-mediated gene transfer on evolutionary dynamics.

As a gene transfer mechanisms, vesicle mediated transfer is surprisingly similar to transduction in many aspects. Harvested P1 phage is estimated to contain 10^9^ to 10^10^ plaque-forming units/mL^93^, as compared to 10^9^ vesicles/mL. Approximately 0.1% of P1 phage particles contain transducing regions^94–97^, whereas we measured as low as 4% packing of plasmids in vesicles. To transfer a genomic region through P1 transduction typically takes several hours^94^, and vesicle-mediated transfer occurs over a few hours. It is not yet clear if transduction and vesicle-mediated transfer are equally prevalent mechanisms of gene transfer, and further quantitative comparisons between the rates and specificity of transduction and vesicle-mediated transport are warranted.

Vesicle-mediated transfer overcomes barriers observed in other forms of gene transfer. Fulsundar *et al*. recently reported the ability of EVs from *A*. *baylyi* to facilitate interspecies gene exchange to *E*. *coli* ^23^. In our study, we expand on this observation by extensively investigating the scope of vesicle-mediated interspecies gene exchange using three donor bacterial species and five recipient species. Plasmid identity exhibited a greater effect on DNA packaging into EVs than the species origin of vesicle production (Figs 1D and 3D). The donor and recipient species also seem to set the overall transfer rate. Some species, such as *A*. *veronii*, make vesicles that facilitate HGT better than the other two donor species tested. A similar trend is seen with the identity of the recipient species, with *P*. *aeruginosa* taking up resistance genes from all three donors strains faster than the first measurement. Although the mechanism that leads to one species being a better donor than another is unknown, specific characteristics of some species confer a greater capacity to transfer DNA via EVs. Future work to identify bacterial features that can affect the ability of particular vesicles to mediate DNA exchange more readily and efficiently would be important to better understand gene exchange patterns in the wild.

Global patterns of interspecies gene exchange have shown that relatedness strongly influences gene transfer rates. Gene maintenance and expression subsequent to transfer contribute to successful HGT and scale with relatedness^17, 85^. However, the initial mechanism to transport genes between cells also contribute to patterns of interspecies exchange. Transduction and conjugation depend on the relatedness of the donor and recipient species^17, 85^. Here we found no clear correlation between relatedness of the donor and recipient and transfer rates. The two recipients strains that were the most distantly related had the slowest and fastest uptake rates, *C*. *violaceum* and *P*. *aeruginosa* respectively. All the bacteria used in this study were proteobacteria, and future work should examine the possibility for vesicle-mediated gene exchange between more diverse species. However, given that in other contexts vesicles enable molecular exchange between very distantly related organisms including bacteria and eukaryotic hosts^13, 15, 16, 18^, it seems plausible that EVs strongly contribute to gene exchange patterns in natural communities, particularly exchange of non-specialized genetic material between distantly related bacteria. Vesicles also serve to protect genetic material from degradation, which should increase both the rate and spatial range of gene exchange^69, 71^. Alves *et al*. demonstrated enzymes protected in EVs maintain enzyme activity^65^. Quantifying the dynamics of gene transfer via vesicles in mixed populations of multiple donor and recipient strains would aid in understanding gene flow within diverse bacterial communities.

## Materials and Methods

### Bacterial Strains and Growth Conditions

All bacterial strains used are listed in Table S2 ^95–97^. Bacteria were grown in Luria-Bertani (LB) broth (Difco, Sparks, MD) at 37** °**C with shaking at 200 rpm. Antibiotics were added to liquid cultures as needed for plasmid maintenance. *A*. *veronii*, *E*. *cloacae* and *E*. *coli* were transformed by electroporation with plasmids listed in Table S1. Following transformation, *A*. *veronii*, *E*. *cloacae* and *E*. *coli* were grown on LB agar plates containing either 35 μg ml^−1^ chloramphenicol, 50 μg ml^−1^ kanamycin or 50 μg ml^−1^ carbenicillin.

### Isolation and purification of EVs

EVs were isolated from liquid cultures of *A*. *veronii*, *E*. *cloacae* and *E*. *coli* as previously described^81^ with some modifications. 400 μl of overnight culture was used to inoculate 400 ml of LB broth containing selective antibiotic. Liquid cultures were grown at 37 °C with shaking at 200 rpm for 16–20 h. Cells were pelleted by centrifugation at 1,200 × g at 4 °C for 30 min. The supernatants were decanted and vacuum filtrated through ExpressPlus 0.45 μm pore-size polyethersulfone (PES) bottle top filter (Millipore, Billerica, MA) to remove remaining cells and cellular debris. Vesicles were collected from the supernatant of 400 ml of liquid culture by ultra-centrifugation at 60,000 × g (Ti 45 rotor; Beckman Instruments, Inc., Fullerton, CA) at 4 °C for 1.5–2 h. Pellets were resuspended in 15 ml of PBS followed by an additional centrifugation of the supernatant at 160,000 × g (Ti 70i rotor; Beckman Instruments, Inc., Fullerton, CA) at 4 °C for 1.5–2 h. The pelleted vesicles were resuspended in 1 ml of phosphate buffered saline (PBS) and stored at 4 °C. Vesicle preparations were treated with 100 ng ml^−1^ of DNase I at 37 °C for 20 min followed by deactivation of the DNase at 80 °C for 10 min. Vesicle preparations were also plated on LB agar to check for the presence of bacterial cells. At room temperature it has been reported that vesicles are stable for 1 week and over a month at −20 °C^69^. In our experiments vesicles were used within 1 week of harvesting.

### EV protein concentration

Vesicle concentrations was quantified using SDS-Polyacrylamide gel electrophoresis. Vesicle preparations were treated with 6xSDS loading buffer and boiled for 10 min at 100 °C and run on a 10% SDS-PAGE gel (Bio-Rad Laboratories, Hercules, CA), stained for 15 min with Coomassie Brilliant Blue Stain, and destained in H_2_O, methanol, and acetic acid (50/40/10 v/v/v) overnight. Protein concentrations were determined using ImageJ from a standard curve generated by a BSA protein concentration gradient.

### EV size characterization using Dynamic Light Scattering (DLS)

DLS was used to characterize the size of purified EVs. Purified EVs were analyzed using a Wyatt Technology DynaPro Titan (Wyatt Technology Corp., Santa Barbara, CA) equipped with a 0–50 mW laser at 830 nm as a light source. The scattered photons were detected at 90°.

### Real-Time PCR

DNA concentration in purified EVs was determined using real-time PCR, Bio-Rad DNA Engine Opticon 2 System for Real-Time PCR Detection (Bio-Rad Laboratories, Hercules, CA), using SYBR Green (Thermo Fisher Scientific Inc., Waltham, MA). Briefly, the reaction consisted of 2 μL of EVs, 0.2 μM of primers, and 1 U of Phusion High-Fidelity DNA Polymerase (New England BioLabs Inc., Ipswich, MA) in a final volume of 45 μL. Each of 35 cycles was denatured at 98 °C for 10 s, annealing at 60 °C for 20 s and extension at 72 °C for 15 s.

### EV-mediated gene transfer

Gene transfer experiments were modified from previously published work^7, 25^. The recipient strains, *A*. *veronii*, *C*. *violaceum*, *E*. *cloacae*, *E*. *coli*, and *P*. *aeruginosa*, were grown in 4 mL LB broth (Difco, Sparks, MD) at 37 °C with shaking at 200 rpm to early log phase, OD_600_ 0.2, ~1–2 h. Then at time 0 h, 0.01 mg purified vesicles was added. Every hour, 200 μL of culture was removed and plated on LB agar plates containing either 35 μg ml^−1^ chloramphenicol, 50 μg ml^−1^ kanamycin or 50 μg ml^−1^ carbenicillin dependent on plasmid resistance. 100 μg ml^−1^ kanamycin was used for plating *P*. *aeruginosa*. After 16 h of incubation at 37 °C, plates were counted and scored for CFUs. The bacterial colonies that acquired antibiotic resistance were re-select on antibiotic selection plates and screened for the presence of the plasmid using PCR. Gain of resistance not associated with plasmid transfer was not observed.

### Statistical Analysis

All two-tailed P values were obtained using unpaired *t* test to compare the means with standard deviations of two groups with n > 3.

## Electronic supplementary material

Supplementary Information
