# Supplementary material for: Genetic cargo and bacterial species set the rate of vesicle-mediated horizontal gene transfer
Source: Sci Rep. 2017 Aug 18;7:8813. doi: 10.1038/s41598-017-07447-7 (PMC5562762; doi:10.1038/s41598-017-07447-7)
Supplement: Supplementary file 1 — Supplementary Information [file 41598_2017_7447_MOESM1_ESM.pdf]

Genetic cargo and bacterial species set the rate of vesicle-mediated horizontal gene transfer

Frances Tran and James Q. Boedicker

Supplementary Information

Table of Contents

Supplementary Table 1: Plasmids Used

Supplementary Table 2: Bacterial Strains

Supplementary Table 3: List of Oligonucleotide Primers Used

Supplementary Figure 1: *E. coli* EVs load different plasmids.

Supplementary Figure 2: BSA protein standard for vesicle protein quantification.

Supplementary Figure 3: Protein assay for vesicle protein quantification.

Supplementary Figure 4: Relative vesicle production measured by lipophilic dye.

Supplementary Figure 5: Calculating plasmid copy number and load per vesicle.

Supplementary Figure 6: Impact of the resistance marker on EV-mediated gene transfer.

Supplementary Figure 7: EV-mediated transfer time for plasmids with different origins.

Supplementary Figure 8: Recovery time after electroporation for the five recipient species.

Supplementary Text 1: Calculating the vesicle number.

Supplementary Text 2: Estimating the time needed for vesicle-mediated gene transfer.

Supplementary Table 1. Plasmids Used

Plasmid	Origin	Size (bp)	Copy	Antibiotic	Original Host	Source
pLC291	RK2	7506	300-400	kanamycin	*Klebsiella aerogenes*	Plasmid #44448 Addgene, Cambridge, MA
pUC19	pMB1	2686	500-700	ampicillin	*E. coli*	#N3041 NEB, Ipswich, MA
pZS2501	SC101	4299	~10	chloramphenicol	*E. coli*	Boedicker Lab [1]

Supplementary Table 2. Bacterial Strains

Species	Strain	Source
Aeromonas veronii	Wild Isolate	Guo *et al.* 2016 [2]
Chromobacterium violaceum	Item #154931A	Carolina Biological, Burlington, NC
Eneterbacter cloacae	Wild Isolate	Souza *et al.* 1999 [3]
Escherichia coli	MG1655	Boedicker *et al.* 2013 [1]
Pseudomonas aeruginosa	UCBPP-PA14	Kreamer *et al.* 2015 [4]

Supplementary Table 3. List of Oligonucleotide Primers Used

Primer	Sequence
pLC291 forward primer	5’-CGTAATGCTCTGCCAGTGTT-3’
pLC291 reverse primer	5’-ATTCAACGGGAAACGTCTTG-3’
pLC291 qPCR forward primer	5’-CCAAGCTAGCAGGGAGAGA-3’
pLC291 qPCR reverse primer	5’-GCCAATACAATGTAGGCTGCTC-3’
pUC19 forward primer	5’-CCCAGTCACGACGTTGTAAAACG-3’
pUC19 reverse primer	5’-AGCGGATAACAATTTCACACAGG-3’
pUC19 qPCR forward primer	5’-AGCGTCAGACCCCGTAGAA-3’
pUC19 qPCR reverse primer	5’-CGGCTACACTAGAAGAACAG-3’
pZS2501 forward primer	5’-GCCCGAAGGTTATGTACAGG-3’
pZS2501 reverse primer	5’-CTTTTCGTTGGGATCTTTCG-3’
pZS2501 qPCR forward primer	5’-TTTCTGTCAGTGGAGAGG-3’
pZS2501 qPCR reverse primer	5’-TACATAACCTTCGGGCATGG-3’

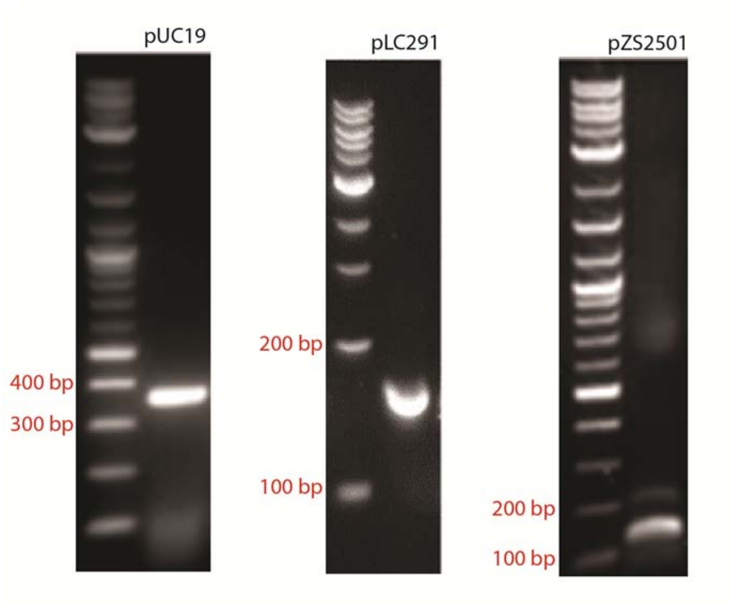

Supplementary Figure 1. *E. coli* EVs load different plasmids, pUC19, pLC291, and pZS2501. PCR targeting each plasmid is performed on EVs isolated from *E. coli* liquid culture transformed with one of three plasmids**.** DNA gel shows PCR products with expected lengths: pUC19 product ~380bp, pLC291 product ~160bp, and pZS2501 product ~120bp.

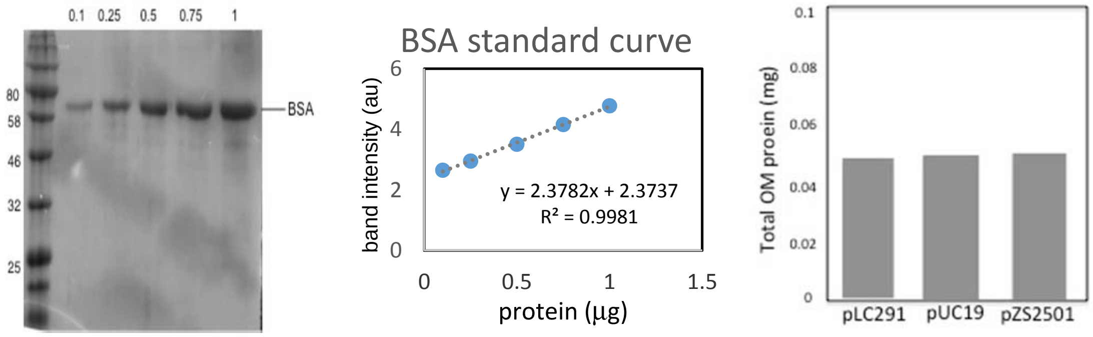

Supplementary Figure 2. BSA protein standard for vesicle protein quantification. Purified BSA protein standard concentrations (0.1µg, 0.25µg, 0.5µg, 0.75µg, and 1µg) were run on a 10% SDS-PAGE gel and stained with Coomassie Blue. Protein concentrations were quantified and plotted using ImageJ and graphed.

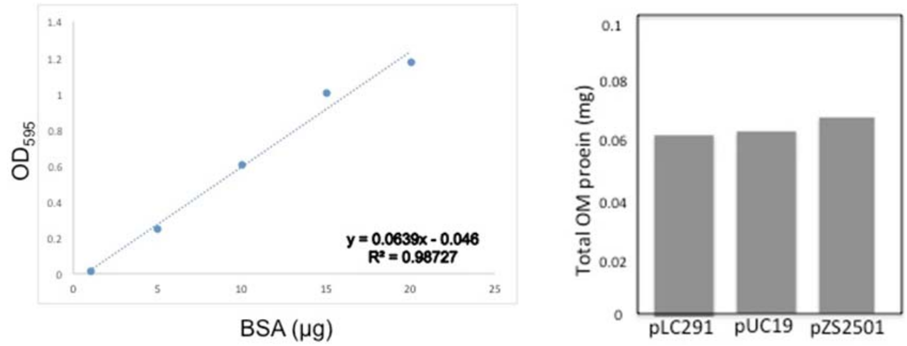

Supplementary Figure 3. Protein Assay for vesicle protein quantification. Purified BSA protein standard concentrations (0.1μg, 0.25μg, 0.5μg, 1μg, and 1.2μg) mixed with BioRad protein assay dye were measured using a spectrophotometer at OD 595. A BSA standard curve was generated and total protein concentration was calculated and graphed.

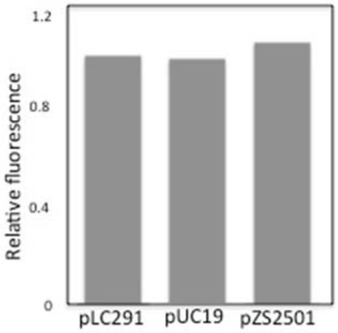

Supplementary Figure 4. Relative vesicle production. The relative quantities of EVs were measured using the fluorescent lipophilic dye FM4-64.

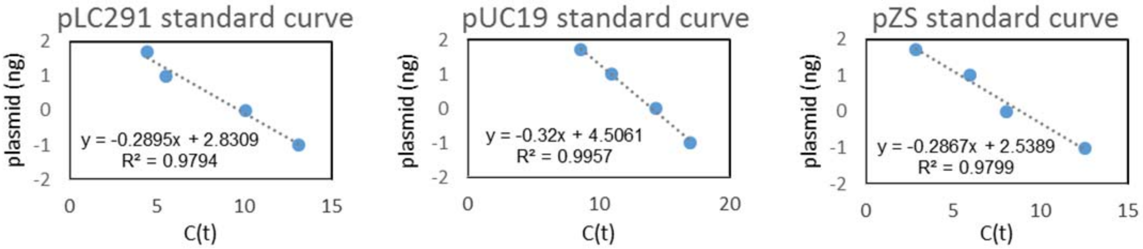

Supplementary Figure 5. Calculating plasmid copy number and load per vesicle. Quantitative PCR standard curves. Standard curves were generated from quantitative PCR of purified plasmid prep of pLC291, pUC19 and pZS2501. Using the standard curve, plasmid concentration per vesicle protein concentration was determined and converted into copy number using the equation above. Number of copies was calculated using: Number copies (ng*6.022x10^23)/(length*1x10^9*650). Subsequently, plasmid loading per vesicle was estimated from reported outer membrane protein concentrations [5] of 6 x 10^{-9} μg OmpA per μm^2 and an average vesicle diameter of 0.2 μm.

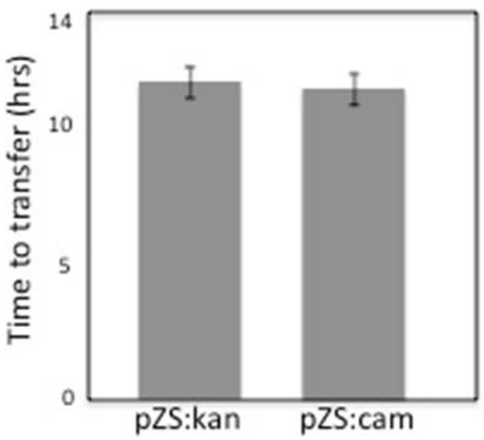

Supplementary Figure 6. EV-mediate transfer was performed using *E. coli donor* EV*s* loaded with pZS conferring either kanamycin or chloramphenicol resistance. Time to transfer is measured by the time to appearance of the first colony forming unit conferring antibiotic resistance. P-value=0.6213

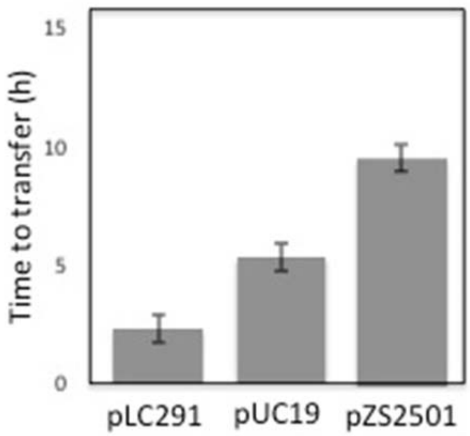

Supplementary Figure 7. EV-mediated transfer was performed using *E. coli* donor EVs loaded with 3500 bp plasmids with one of three origins of transfer, RK2, colE1, or SC101. Time to transfer is measured by the time to appearance of the first colony forming unit conferring antibiotic resistance. All pairs of transfer times have p-values <0.05.

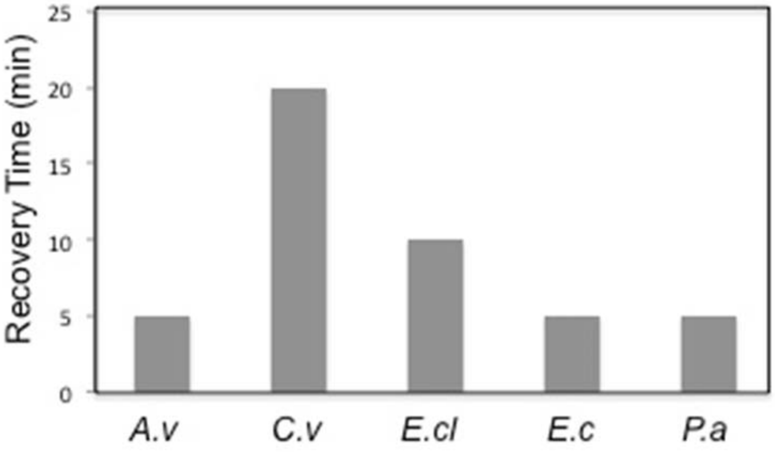

Supplementary Figure 8. Transformation by electroporation was performed using pUC19 conferring ampicillin resistance into each of five bacterial species, *A. veronii*, *C. violaceum*, *E. cloacae*, *E. coli*, and *P. aeruginosa*. The minimum time needed to recover after electroporation was measured by plating on LB agar plates with ampicillin.

Calculating the vesicle number:

The density of vesicles harvested from donor cell cultures was estimated from the measurements of outer membrane protein concentrations and light scattering measurements of vesicle size. Omp, Omp, and Omp are the major protein component of a bacterial membrane, and are used as a proxy for the total amount of lipid harvested from donor cultures [6-8].

To calculate the area of lipid (A_{lipid}), we use:

$$A_{lipid} = m_{omp}\, 6 \times 10^{-9}\ \mu g\ OmpA/\mu m^2, \qquad \text{(Equation S1)}$$

where m_{omp} is the mass of Omp A protein from the protein gel in µg. Reference 92 in the main text reports that *E. coli* has 6×10^{-9} µg OmpA/µm^2.

To convert the area of lipid to the number of vesicles, we use:

$$N_{ev} = A_{lipid} / (\pi\, d_{ev}^2), \qquad \text{(Equation S2)}$$

where d_{ev} is the average diameter of an extracellular vesicle. Here we use 0.2 µm as the average diameter of all vesicles in our study, in agreement with dynamic light scattering measurements reported in Figure 1D. From these calculations we find on the order of 6×10^{11} vesicles were harvested from 400 mL of stationary phase culture.

Estimating the time needed for vesicle-mediated gene transfer

Given that this amount of vesicles was harvested from 400 mL of stationary phase culture, if we assume no loss of vesicles during isolation we have approximately,

6×10^{11} vesicles / (400 mL * 4×10^9 cells/mL) = 0.4 vesicles/cell. (Equation S3)

0.4 vesicles per cell, although a crude estimate, is close to reported numbers of 2-5 vesicles produced per cell per generation of *Prochlorococcus*[47]. Although vesicles are constantly produced, degraded, and taken up within the culture, it does suggest in our growth condition we should expect approximately equal numbers of cells and vesicles.

For transfer experiments, we calculate 1.3×10^{10} EVs were added to 4 mL of culture. This would give:

1.3×10^{10} vesicles / (4 mL * 4×10^9 cells/mL) = 0.8 vesicles/cell. (Equation S4)

Therefore, vesicle uptake by recipient cells was measured at a ratio of vesicles to cells only a factor of 2 higher than estimated for stationary phase cultures.

From Figure 2C, we found that a factor of 10 reduction in the number of vesicles resulted in about a 1.5 hour increase in the transfer time. Although not tested, if we assume a 10 fold change in either vesicle number or recipient cell number result in similar delays in transfer time, we could estimate how long it would take to observe transfer between two stationary phase cultures with a volume of 1 mL. 1 mL reduces the cell number by a factor of 4 as compared to the 4 mL cultures used in the transfer experiments. The number of vesicles produced by 1 mL of stationary phase culture is 1.6×10^9 vesicles (based on 0.4 vesicles/cell calculated above), or an 8 fold reduction in vesicle number. Together, that would be equivalent to a 32 fold reduction in vesicles. Based on Figure 2C, this suggests a single vesicle transfer would occur within 1 mL of stationary phase culture every 7.3 hours. These are very rough calculations, but does suggest that vesicle mediated gene transfer would be occurring within populations of a billion cells over time scales less than a day. As shown in Figure 2B and 4B-D, the plasmid transferred and the donor and recipient strains also modulate the vesicle transfer times by multiple hours. Additionally it is known that culturing conditions and cell stress both influence vesicle production. It is likely that exchange times under a specific set of circumstances could be anywhere from 1 hour to multiple days. More experimental work is needed to further quantify vesicle exchange times per cell under a variety of conditions.

Supplemental References:

1. Boedicker, J. Q., Garcia, H. G. & Phillips, R. Theoretical and Experimental Dissection of DNA Loop-Mediated Repression. *Phys. Rev. Lett.* **110,** 18101 (2013).
2. Guo, X. & Boedicker, J. Q. The Contribution of High-Order Metabolic Interactions to the Global Activity of a Four-Species Microbial Community. *PLoS Comput. Biol.* **12,** e1005079 (2016).
3. Souza, V., Rocha, M., Valera, A. & Eguiarte, L. E. Genetic structure of natural populations of Escherichia coli in wild hosts on different continents. *Appl. Environ. Microbiol.* **65,** 3373–85 (1999).
4. Kreamer, N. N., Phillips, R., Newman, D. K. & Boedicker, J. Q. Predicting the impact of promoter variability on regulatory outputs. *Sci. Rep.* **5,** 18238 (2015).

5. Aldea, M., Herrero, E., Esteve, M. I. & Guerrero, R. Surface density of major outer membrane proteins in Salmonella typhimurium in different growth conditions. *J. Gen. Microbiol.* **120,** 355–67 (1980).
